# Evaluation of Safety and Immunogenicity of High-Dose Quadrivalent Seasonal Influenza Split Vaccine: A Preclinical Study

**DOI:** 10.3390/vaccines14050446

**Published:** 2026-05-17

**Authors:** Lanxin Jia, Ran Qiu, Jing Liu, Bo Liu, Xuanxuan Nian, Yang Le, Xixin Han, Qingmei Zhang, Xuedan Li, Zheng Gong, Ailin Shen, Zhegang Zhang, Ying Zhao, Jiayou Zhang

**Affiliations:** 1 Wuhan Institute of Biological Products Co., Ltd., Wuhan 430022, China; lanxinj2020@163.com (L.J.); qiuran1107@163.com (R.Q.); zhangzhegang@sinopharm.com (Z.Z.); 2 National Engineering Technology Research Center for Combined Vaccines, Wuhan 430022, China; 3 National Key Laboratory for Novel Vaccines Research and Development of Emerging Infectious Diseases, Wuhan 430022, China; 4 Hubei Provincial Vaccine Technology Innovation Center, Wuhan 430022, China; 5 Department of Pharmacy, Union Hospital, Tongji Medical College, Huazhong University of Science and Technology, Wuhan 430022, China

**Keywords:** high-dose quadrivalent influenza vaccine, safety, immunogenicity, preclinical study, aged mice

## Abstract

Objectives: Seasonal influenza leads to substantial global morbidity and mortality, especially in adults aged 65 years and older, who present poor immune responses to standard-dose influenza vaccines. This study aimed to systematically evaluate the preclinical safety and immunogenicity of a high-dose quadrivalent seasonal influenza split vaccine (HD-QIV), providing preclinical evidence for its clinical application in the elderly. Methods: Following GLP guidelines, we performed single-dose and repeated-dose toxicity tests in Sprague–Dawley rats, active systemic anaphylaxis assays in guinea pigs, and immunogenicity assessments in young and aged BALB/c mice. Safety indicators included general clinical signs, hematology, blood biochemistry, histopathology and allergic reactions; immunogenicity was evaluated via hemagglutination inhibition (HI) antibody titers and antigen-specific cellular immune responses. Results: HD-QIV only caused mild and reversible local irritation in rats without obvious systemic toxicity, and no dose-related systemic anaphylaxis was observed in guinea pigs. HD-QIV induced robust and dose-dependent humoral immune responses, and showed significantly higher HI antibody titers, earlier seroconversion and longer antibody persistence than standard quadrivalent influenza vaccine in aged mice. Cellular immunity was slightly enhanced but not the dominant protective response. Conclusions: The HD-QIV demonstrates favorable preclinical safety and superior immunogenicity, supporting its further clinical development and use as a priority vaccine for the elderly population.

## 1. Introduction

Seasonal influenza virus can infect 5–15% of the global population annually, cause 3–5 million cases of severe illness, and lead to up to 500,000 deaths from influenza and its complications, making it one of the major public health challenges globally [1,2,3]. Adults aged 65 years and older are much more likely to develop severe complications after influenza infection than other age groups, accounting for the majority of severe illnesses and deaths associated with seasonal influenza, which may be related to declining immune responses and underlying diseases [4,5]. Vaccination is the most effective strategy for interrupting the spread of influenza viruses in the population and preventing severe influenza [6]. However, according to the Centers for Disease Control and Prevention (CDC), the protection rate of standard-dose influenza vaccine in people over 65 years old during the past 10 years is less than 60%, and as low as 20% in some epidemic seasons, which is far from satisfactory for protecting this vulnerable group. This suboptimal protection can be attributed to multiple critical factors: age-associated immunosenescence, insufficient antigen content, impaired antigen presentation, and antigenic mismatch between predicted vaccine strains and circulating influenza viruses [7,8]. Therefore, improving the efficacy of influenza vaccines in the elderly population has been a critical and active area of research.

Fluzone is a high-dose inactivated influenza vaccine developed by Sanofi Pasteur, which contains four times the amount of hemagglutinin per strain as SD-IIV [9]. High-dose trivalent inactivated influenza vaccine (HD-IIV) was initially approved by the Food and Drug Administration (FDA) in 2009 for adults aged 65 years and older. Over the following decade, sustained co-circulation of two influenza B lineages in the population prompted the approval of the high-dose quadrivalent inactivated influenza vaccine (HD-QIV) in 2019 [10,11]. Findings from a real-world randomized study involving 45 million participants across 12 influenza seasons (2009/2010 to 2019/2020, 2021/2022) indicated that, in adults aged ≥65 years, HD-IIV confers significantly greater protection against influenza-like illness and influenza-associated hospitalization, independent of circulating strains and antigenic match [12,13,14,15]. Furthermore, multiple studies have shown that high-dose influenza vaccines have a favorable safety profile: a systematic review indicated that no serious adverse reaction occurred after administration of the HD-IIV [16], and recent three-year post-marketing surveillance of HD-QIV conducted in Finland and Germany similarly found no relevant safety issues [17,18,19]. The Advisory Committee on Immunization Practices (ACIP) recommends that people aged 65 years and older receive HD-QIV, quadrivalent recombinant influenza vaccine (RIV4), or adjuvanted quadrivalent inactivated influenza vaccine (aIIV4) as a priority [20]. In an Italian elderly population (≥65 years), HD-QIV was more cost-effective than aIIV4, generating better health outcomes at an acceptable incremental cost or even cost-saving [21].

The seasonality of influenza in China exhibits substantial diversity. Northern and southern regions show influenza peaks in winter and spring, while southern regions present an additional summer peak [22,23]. Surveillance data from the Chinese National Influenza Center indicate that non-pharmaceutical interventions indirectly suppressed national influenza activity during the COVID-19 pandemic. Influenza prevalence remained extremely low in 2020 and has risen gradually alongside the relaxation of public health measures in recent years [24,25,26]. Influenza vaccination coverage in China remains below 5%, representing a substantial gap compared to developed countries. Meanwhile, China is undergoing rapid population aging, with official statistics indicating that the population aged 65 and above totals 220.23 million, accounting for 15.6% of the national population. This sizeable elderly susceptible population is expected to impose a heavy burden on China’s healthcare system. Therefore, based on the existing production platform for quadrivalent inactivated influenza vaccines, this study further developed a HD-QIV and evaluated its safety and immunogenicity in strict adherence to the requirements of Good Laboratory Practice (GLP) for Non-Clinical Studies, aiming to provide a preclinical basis for future influenza prevention and control efforts targeting the elderly.

## 2. Materials and Methods

### 2.1. Animals & Ethics Statement

Sprague–Dawley (SD) rats (6–7 weeks of age) were used to evaluate single- and repeat-dose toxicity studies, and were supplied by Hunan SJA Laboratory Animal Co., Ltd. (Changsha, China). Dunkin Hartley guinea pigs—with males aged 28–49 days and females aged 21–35 days—were obtained from Hunan Taiping Biotechnology Co., Ltd. (Yiyang, China) for the active systemic anaphylaxis assay. For immunogenicity analyses, 6–8 week-old BALB/c mice were provided by Wuhan Institute of Biological Products (Wuhan, China), while 16-month-old BALB/c mice were supplied by Biocytogen Pharmaceuticals (Beijing) Co., Ltd. (Beijing, China). All SD rats and BALB/c mice involved in this preclinical study were raised in specific pathogen-free (SPF) facilities, whereas guinea pigs were raised in conventional (non-SPF) environments. Challenge infection experiments were conducted in an individually ventilated caging (IVC) system.

### 2.2. Vaccine Preparation

The high-dose quadrivalent seasonal influenza split vaccine (HD-QIV) was produced by the Influenza Virus Vaccine Room of Wuhan Institute of Biological Products (Wuhan, China) in accordance with the approved manufacturing and verification protocols. The influenza virus strains recommended by the World Health Organization (WHO) for the 2022/2023 epidemic season—including H1N1 (A/Victoria/2570/2019), H3N2 (A/Darwin/9/2021), B/Victoria lineage (BV, B/Austria/1359417/2021), and B/Yamagata lineage (BY, B/PHUKET/3073/2013)—were inoculated into age-appropriate embryonated chicken embryos. Following incubation at 34 °C for 48–72 h, the virus-containing allantoic fluid was harvested and inactivated with formaldehyde. Subsequently, the fluid underwent ultrafiltration and concentration, purification, and chemical disruption using the non-ionic surfactant octylphenol ethoxylate (Triton^®^ X-100, Sigma-Aldrich, St. Louis, MO, USA) to form the split virus, which was further purified and sterilized by filtration to prepare monovalent vaccine stock solutions. HD-QIV, formulated by mixing the four separately produced monovalent stock solutions, contains 60 μg of hemagglutinin (HA) per strain per 0.7 mL vial. It is free of adjuvants or preservatives and is a slightly milky white liquid. Each vial is labeled with product name, manufacturer, batch number, and expiration date.

### 2.3. Safety Evaluation

#### 2.3.1. Single Dose Toxicity Study in SD Rats

A total of 40 SPF SD rats with a sex ratio of 1:1 (male:female) were randomly assigned to two groups: a negative control (NC) group and an HD-QIV experimental group, with 20 rats per group. The body weights of female and male rats ranged from 195.39 to 217.54 g and 232.74 to 258.93 g, respectively. All rats were acclimated to the environment for at least one week prior to study initiation.

For dosing, rats in the experimental group received 2.1 mL of HD-QIV per animal via multipoint intramuscular injection (bilateral quadriceps femoris or lateral thigh muscle groups, ≥1 cm between injection sites, ≤0.2 mL per site), representing 3 times the clinical inoculation dose and approximately 900 times the clinical weight-based dose. While those in the NC group were injected with an equal volume of 0.9% sterile sodium chloride solution using the same injection method. The dosing day was defined as Study Day 1 (D1).

**Figure 1:**
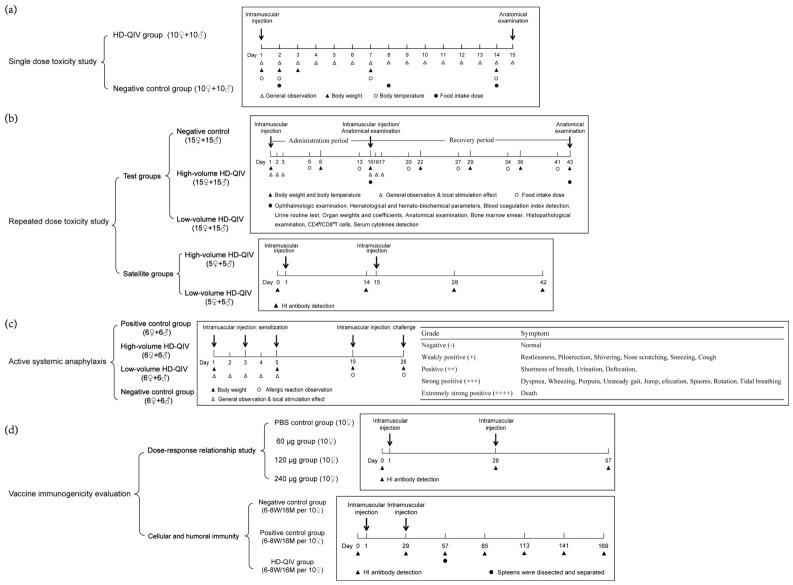
Group design for the safety and immunogenicity evaluation of HD-QIV. (**a**) Single-dose toxicity study design in SD rats; (**b**) Repeated-dose toxicity study design in SD rats; (**c**) Active systemic anaphylaxis study design in guinea pigs; (**d**) Immunogenicity evaluation study design.

Following injection, all animals were monitored daily for 14 consecutive days. Observations included general appearance, physical signs, behavior patterns, food consumption, fur condition, local irritation reactions at injection sites, ocular and nasal secretions, fecal characteristics, respiratory status, and mortality. Body weights were recorded on D1, D2, D3, D7, and D14; body temperatures were measured on D1, D2, D7, and D14; and daily food intake was assessed on D2, D8, and D14. At the end of the observation period (D15), all surviving animals were euthanized and subjected to gross necropsy. The study design, including sampling time points and related measurements, is shown in Figure 1a.

#### 2.3.2. Repeated-Dose Toxicity Assay in SD Rats

A total of 90 SPF SD rats (1:1 male-to-female ratio) were used, with body weights ranging from 190.53 to 216.74 g for females and 234.17 to 255.07 g for males. Rats were randomly assigned to 3 main groups using a random number table (RNT) method: a negative control (NC) group, a HD-QIV low-volume group, and a HD-QIV high-volume group, with 30 rats per group (15 rats of each gender). Two additional satellite groups (HD-QIV low-volume group and HD-QIV high-volume group) were included for recovery observation, with 10 rats per group (5 rats of each gender).

On Study Day 1 (D1) and Day 15 (D15), rats in the HD-QIV low-volume group received 0.7 mL of HD-QIV via multipoint intramuscular injection (bilateral quadriceps femoris or lateral thigh muscle groups, ≥1 cm between injection sites, ≤0.2 mL per site). Rats in the HD-QIV high-volume group received 2.1 mL of HD-QIV using the same procedure, while those in the NC group were injected with 2.1 mL of 0.9% sterile saline. All animals were monitored for 4 weeks after the final injection (Figure 1b).

Daily observations included general health status; body weights, body temperatures, and 24 h food intake were recorded weekly. Ophthalmic examinations (assessing eyelids, conjunctiva, cornea, pupil, and iris) were conducted on D15 and D43 after the first injection. Urine samples were collected using plastic film at designated time points for urinalysis. At the study endpoint, rats were anesthetized via intraperitoneal injection of Zoletil^®^50 (30 mg/kg; Virbac, Carros, France), followed by abdominal aorta blood collection. Whole blood samples were used for hematological tests, coagulation assays, and T lymphocyte subset analysis; serum samples were analyzed for blood biochemistry parameters and hemagglutination inhibition (HI) antibody titers. After fasting and anesthesia, rats were euthanized by exsanguination, followed by gross necropsy. The wet weights of major organs were measured, and organ coefficients were calculated as the ratio of organ weight to body weight. Eyeballs and testes/epididymides were fixed in modified Davidson’s fixative, then transferred to 10% neutral buffered formalin (NBF; Solarbio, Beijing, China) within 24–48 h. All other tissues were directly preserved in 10% NBF. Subsequently, all organ/tissue samples were embedded in paraffin, sectioned, and stained with hematoxylin and eosin (H&E) for histopathological examination.

#### 2.3.3. Systemic Allergy Test in Guinea Pigs

A total of 48 healthy Dunkin Hartley guinea pigs (1:1 male-to-female ratio) were used. Females were 4–6 weeks old (body weight: 310.54–361.21 g) and males were 5–8 weeks old (body weight: 346.82–383.14 g). Guinea pigs were randomly assigned to four groups via body weight stratification: a HD-QIV low-volume group, a HD-QIV high-volume group, a negative control (NC) group, and a positive control group.

All animals received three intramuscular sensitizing injections at one-day intervals. The low- and high-volume groups were given 0.07 mL and 0.7 mL of HD-QIV per injection, whereas the NC group was given 0.7 mL of 0.9% sterile saline and the positive control group 0.7 mL of 1% ovalbumin. At Days 14 and 21 after the last sensitization, all guinea pigs were challenged via intravenous injection with a two-fold dose of the respective sensitizer: 0.14 mL of HD-QIV for the low-volume group, 1.4 mL of HD-QIV for the high-volume group, 1.4 mL of 0.9% sterile saline for the NC group, and 1.4 mL of 1% ovalbumin for the positive control group.

Two additional groups of non-sensitized healthy guinea pigs (4 animals per group, 2 males/2 females) were included to verify whether the observed reactions were anaphylactoid. If allergic-like symptoms occurred in the low-volume or high-volume groups post-challenge, one male and one female guinea pig from each verification group were intravenously injected with the same challenge dose of HD-QIV for confirmation.

During the sensitization phase, general health status was monitored daily. Body weights were recorded on the day of the first sensitization, the last sensitization, and each challenge. Post-challenge, detailed observations of each animal’s responses were conducted—including the onset and resolution of symptoms—and reactions were graded based on a standardized symptom scale (Figure 1c).

### 2.4. Vaccine Immunogenicity Analysis

#### 2.4.1. Animal Immunization

SPF female BALB/c mice (6–8 weeks old) were randomly assigned to 4 groups (*n* = 10) and immunized via multipoint intramuscular injection with QIV at 28-day intervals (two immunizations total). The inoculation volume was 0.7 mL per mouse, administered into the bilateral quadriceps femoris or lateral thigh muscle groups, ≥0.5 cm between injection sites, ≤0.2 mL per site to minimize local tissue irritation. Total antigen contents were 60 μg, 120 μg, and 240 μg (15 μg, 30 μg, and 60 μg per antigen subtype, respectively); an equal volume of phosphate-buffered saline (PBS) served as the negative control, delivered via the same multipoint injection approach. Blood samples were collected before immunization (baseline) and on Days 28 and 56 after the primary immunization.

The antigen dose–response study in SD rats was conducted concurrently with the repeated-dose toxicity assay (Section 2.3.2). Blood samples from SD rats were collected at baseline (pre-immunization) and on Days 14, 28, and 42 after the first immunization. Hemagglutination inhibition (HI) antibody titers were measured in all serum samples.

In a separate experiment, SPF female BALB/c mice (aged 6–8 weeks and 16 months) were randomly assigned to 3 groups (*n* = 10) and immunized with HD-QIV via the same multipoint intramuscular injection procedure at 28-day intervals. Briefly, the vaccine was administered into the bilateral quadriceps femoris or lateral thigh muscle groups, with injection sites spaced ≥0.5 cm apart and ≤0.2 mL per site. A licensed quadrivalent influenza virus split vaccine (total antigen content: 60 μg, 15 μg per subtype; batch no.: 202209031A) served as the positive control, and 0.7 mL PBS served as the negative control—both delivered using the identical multipoint method. Blood samples were collected on Days 28, 56, 84, 112, 140 and 168 after primary immunization to detect the antibody levels of HI, and the spleens were isolated on Day 56 after the primary immunization to detect the immune cell typing and the level of cells secreting IFN-γ, TNF-α and IL-2 in the spleen (Figure 1d). Table S1 briefly summarizes all experimental groups and key endpoint indicators involved in this study.

#### 2.4.2. Hemagglutination Inhibition Test (HI)

Sera were mixed with receptor-destroying enzyme (RDE; Merck, Darmstadt, Germany) and incubated overnight at 37 °C to eliminate nonspecific hemagglutination inhibitors. After incubation, the sera were heated at 56 °C for 30 min to inactivate RDE activity. Treated sera were serially diluted two-fold with PBS. Influenza virus vaccine strains (4 hemagglutinating units [HAU]/well) were then added to the diluted sera, and the mixture was incubated at room temperature for 30 min. Subsequently, 1% chicken red blood cell suspension was added, and the mixture was incubated for an additional 30 min at room temperature. The HI titer was recorded as the highest dilution of the sera that completely inhibited hemagglutination. A ≥4-fold increase in HI titer from baseline was defined as seroconversion.

#### 2.4.3. Flow Cytometry Analysis

To characterize antigen-specific cellular immune responses, mouse spleens were homogenized in PBS to prepare single-cell suspensions. The suspensions were centrifuged, and the resulting cell pellets were resuspended in RPMI–1640 medium (Gibco, Waltham, MA, USA). Mouse lymphocyte separation medium (DAKEWE, Shenzhen, China) was gently added to the resuspended cell suspensions, and the mixtures were centrifuged to isolate splenic lymphocytes. Isolated lymphocytes were washed with PBS, counted, and adjusted to a final concentration of 1 × 10^6^ cells/well. They were then co-incubated with an influenza virus antigen mixture (HD-QIV used in this study) at a final concentration of 5 μg/mL for 18 h. Brefeldin A (10 μg/mL; BioLegend, San Diego, CA, USA) was added to the cultures, which were incubated for an additional 5 h.

Cells were stained for viability using the Zombie Aqua™ Fixable Viability Kit (BioLegend, San Diego, CA, USA; Cat. No. 423102) for 15 min at room temperature in the dark. After staining, cells were washed with Cell staining buffer (BioLegend, San Diego, CA, USA; Cat. No. 420201) and centrifuged at 300× *g* for 5 min. Surface staining was performed by adding APC/Cyanine7 anti-mouse CD3ε Antibody (BioLegend, San Diego, CA, USA; Cat. No. 100330), FITC anti-mouse CD4 Antibody (BioLegend, San Diego, CA, USA; Cat. No. 100405), and Brilliant Violet 421™ anti-mouse CD8a Antibody (BioLegend, San Diego, CA, USA; Cat. No. 100737), followed by incubation at room temperature in the dark for 30 min. Cells were then washed with Cell staining buffer and centrifuged again (300× *g* for 5 min).

Each sample tube received 500 μL of Fixation Buffer (BioLegend, San Diego, CA, USA; Cat. No. 420801) to fix the cells, which were incubated at room temperature in the dark for 20 min. After fixation, cells were washed with Cell staining buffer, centrifuged (300× *g* for 5 min), and resuspended in 1 mL of Intracellular staining perm buffer (BioLegend, San Diego, CA, USA; Cat. No. 421002) for permeabilization. Following centrifugation (300× *g* for 5 min), intracellular staining was conducted by adding PE/Cyanine7 anti-mouse IL-2 Antibody (BioLegend, San Diego, CA, USA; Cat. No. 503831), PE anti-mouse IFN-γ Antibody (BioLegend, San Diego, CA, USA; Cat. No. 505807), and APC anti-mouse TNF-α Antibody (BioLegend, San Diego, CA, USA; Cat. No. 506308). Cells were incubated at room temperature in the dark for 30 min, then washed sequentially with perm buffer and Cell staining buffer (300× *g* for 5 min per wash). Cells were analyzed using a Beckman CytoFLEX S flow cytometer, and data were processed with FlowJo 10.8 software. The gating strategy is shown in Supplementary Figure S1.

### 2.5. Statistical Analysis

All statistical analyses were performed using GraphPad Prism 10.1.2 software. Data are presented as mean ± SD. Statistical significance was denoted as follows: ns (*p* ≥ 0.05), * (*p* < 0.05), ** (*p* < 0.01), *** (*p* < 0.001), and **** (*p* < 0.0001). Normality was tested by the Shapiro–Wilk test, and homogeneity of variance was evaluated using the Brown-Forsythe test. HI antibody titers were log_2_-transformed prior to between-group comparisons. For safety parameters including body weight, body temperature, and food intake, two-way repeated-measures ANOVA was applied. Mauchly’s test was used to examine the sphericity assumption. When sphericity was violated, the Greenhouse–Geisser correction was adopted, and the Bonferroni post hoc test was used for pairwise comparisons between groups and across time points. For hematology, coagulation indices, serum biochemistry, organ weights, and organ coefficients, one-way ANOVA with Tukey’s HSD post hoc test was used for data with normal distribution and homogeneous variance. For non-normally distributed or heteroscedastic data, the Kruskal–Wallis H test followed by Dunn’s post hoc test with Bonferroni correction was performed. For immunological evaluation, log_2_-transformed HI antibody titers were compared using one-way ANOVA or the Kruskal–Wallis test. Frequencies of immune cell subsets and cytokine-positive cells determined by flow cytometry were analyzed using appropriate parametric or nonparametric tests. A false discovery rate (FDR) correction (Benjamini–Krieger–Yekutieli method) was applied for multiple time-point comparisons.

## 3. Results

### 3.1. No Significant Adverse Effects Were Observed in SD Rats After a Single Dose of HD-QIV

To evaluate the potential acute toxicity of intramuscularly administered HD-QIV, SPF SD rats were assigned to either the NC group or the experimental group. Rats in the experimental group received three doses of HD-QIV intramuscularly within 24 h.

**Figure 2:**
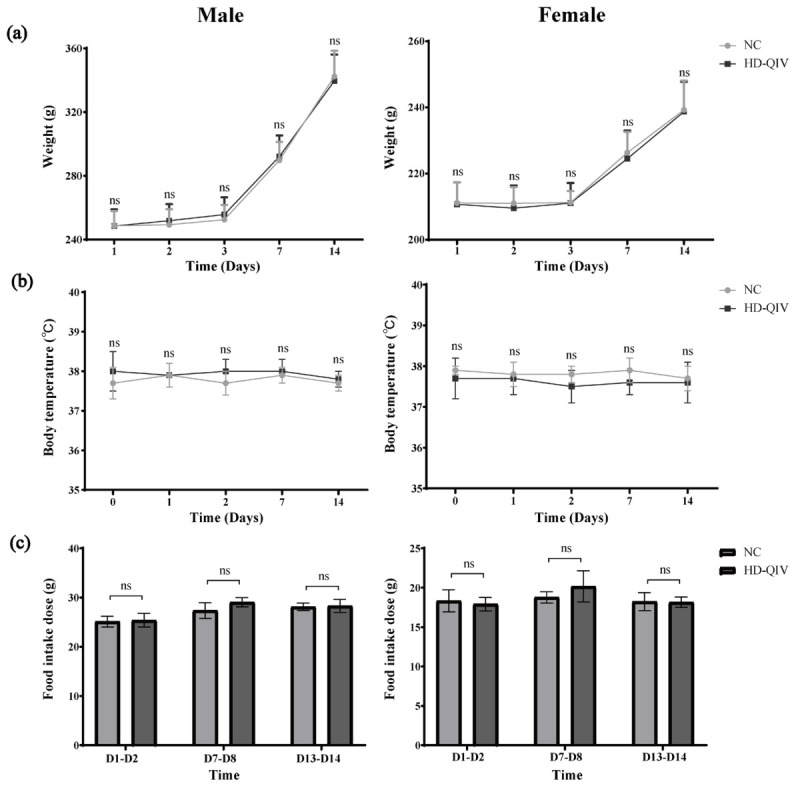
No significant changes in body weight, body temperature, or food intake were observed in the single-dose toxicity study (*n* = 10). (**a**) Body weight of SD rats on Days 1, 2, 3, 7, and 14 after single dose administration; (**b**) Body temperature of SD rats before administration and on Days 1, 2, 7, and 14 after single dose administration; (**c**) Daily food intake of SD rats on Days 2, 8, and 14 after single dose administration. Data are presented as the mean ± SD. ns, *p* ≥ 0.05.

All rats survived the entire experimental period and exhibited normal growth throughout the study. In the HD-QIV group, transient hind limb swelling was observed in 5/10 males and 8/10 females on Day 1, which resolved by Day 3. No significant abnormal changes were observed in body weight, weight gain, food intake, body temperature, or gross anatomy of male or female SD rats in either the experimental group or the negative control group (Figure 2). In conclusion, a single intramuscular injection of HD-QIV at three times the clinically proposed dose (2.1 mL per rat, 180 μg HA per strain) within 24 h had no significant adverse effects in SD rats, except for mild local irritative reactions in some individuals.

### 3.2. No Significant Adverse Effects Were Observed in SD Rats After Repeated Inoculations of HD-QIV

To further characterize the potential irritancy and severity at the administration site, assess HD-QIV-induced toxic reactions, and identify potential toxic target organs, SD rats received HD-QIV intramuscularly once weekly for two consecutive weeks at two dose levels: a low-volume dose equivalent to clinical usage, and a high-volume dose set as a toxicological marginal dose that far exceeds the intended human clinical dose.

**Figure 3:**
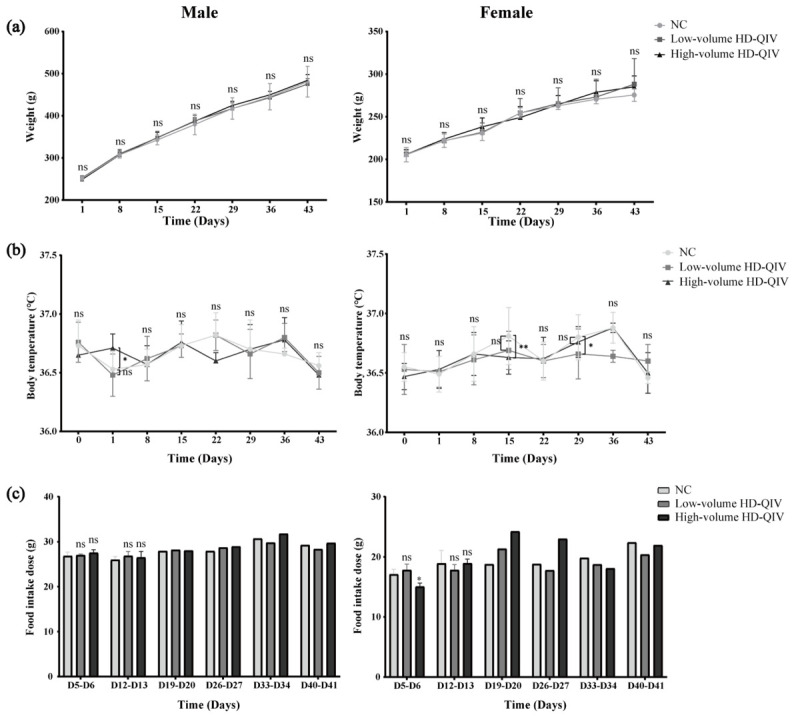
Body weight, body temperature, and food intake in male and female SD rats during HD-QIV repeated-dose toxicity assay. (**a**) Body weight measured on Days 1, 8, 15, 22, 29, 36, and 43 (*n* = 15 for the administration period, *n* = 5 for the recovery period); (**b**) Body temperature assessed before first administration and on Days 1, 8, 15, 22, 29, 36, and 43 (*n* = 15 for the administration period, *n* = 5 for the recovery period); (**c**) Daily food intake recorded on Days 6, 13, 20, 27, 34, and 41 (*n* = 3 for the administration period, *n* = 1 for the recovery period). Data are presented as mean ± SD. ns, *p* ≥ 0.05; *, *p* < 0.05; **, *p* < 0.01.

No HD-QIV-associated mortality was recorded during the 2-week repeated toxicity observation period. Transient unilateral or bilateral swelling at the hind-limb injection sites was observed and resolved completely within 3 days. No notable differences in ophthalmological parameters were detected among all groups. No obvious abnormal changes in body weight were found in any HD-QIV group compared with the negative control group (Figure 3a). Isolated fluctuations in body temperature were observed at individual time points: male rats in the high-volume group showed elevated temperature on Day 1 (*p* < 0.05), and female rats in the high-volume group showed reduced temperature on Day 15 (*p* < 0.05). No other significant changes in body temperature were detected at any other time points (Figure 3b). Similarly, a mild reduction in food intake was seen in high-volume female rats on Days 5–6 (*p* < 0.05), with no significant differences observed across all other time points (Figure 3c).

Of the 24 routine hematological parameters, at the end of the administration period (D15), female rats in the high-volume group exhibited increased mean corpuscular hemoglobin concentration (MCHC), eosinophil proportion (EO%), and fibrinogen (FIB), along with decreased activated partial thromboplastin time (APTT) (*p* < 0.05). In male rats, the low-volume group showed increased thrombin time (TT) (*p* < 0.01), while the high-volume group had elevated FIB (*p* < 0.05). At the end of the recovery period (D44), high-volume female rats still had increased MCHC and FIB, and low-volume female rats showed elevated FIB (*p* < 0.05) (Table 1 and Table S2).

For serum biochemical parameters, at D15, both low- and high-volume groups showed increased total cholesterol (TCHO) and triglycerides (TG), and decreased creatine kinase (CK) compared with gender-matched negative controls. At D44, high-volume male rats had increased serum albumin (ALB) and albumin–globulin ratio (A/G). High-volume female rats showed decreased CK and A/G, increased chloride (Cl^−^) and globulin (GLO). In the low-volume group, male rats exhibited decreased GLO, and increased UREA and A/G; female rats had decreased creatinine (CREA) and A/G, and increased total protein (TP) and GLO (Table 2 and Table S3).

For urinalysis, no significant changes were observed in any group on D14. On D43, high-volume male rats showed decreased ketone bodies (KET) (*p* < 0.05) (Table S4). Necropsy revealed no obvious changes in organ weights or coefficients in any HD-QIV group at the end of the administration period. At the end of the recovery period, high-volume male rats had decreased heart weight and organ coefficient (*p* < 0.05) (Figure S2a). Scattered mononuclear cell infiltration was observed at injection sites after the administration and recovery periods; occasional focal muscle fiber degeneration and interstitial edema were also noted in the high-volume group. For T lymphocyte subsets, no significant changes were detected in any HD-QIV group compared with gender-matched negative controls on D15. On D43, high-volume female rats showed increased CD3^+^CD4^+^ T cells (*p* < 0.05) and decreased CD3^+^CD8^+^ T cells (*p* < 0.05), with a similar trend in males and a clear dose–response relationship (Figure S2b).

Overall, all statistically significant alterations observed in body temperature, food intake, hematology, biochemistry, urinalysis, organ weights, and local tissue were mild, sex-specific, sporadic, and lacked a consistent dose–response relationship. All fluctuations remained within normal physiological ranges of SD rats and were attributed to inherent biological variability rather than adverse effects related to HD-QIV. Local tissue changes at the injection site were consistent with physical irritation caused by repeated intramuscular injection. The mild alterations in T lymphocyte subsets showed a dose-dependent pattern and were regarded as a mild pharmacological immunostimulatory effect without pathological significance. Collectively, the repeated-dose toxicity study demonstrates that HD-QIV is well tolerated and supports its favorable preclinical safety profile for clinical application.

### 3.3. No Active Systemic Allergic Reaction Was Observed in Guinea Pigs Inoculated with HD-QIV

To assess whether intramuscular administration of HD-QIV induces active systemic anaphylaxis in guinea pigs, animals were sensitized and challenged with two dose levels: a low-volume dose, and a high-volume dose equivalent to the clinical dose for human use. Following sensitization and challenge, allergic symptoms were monitored for 30 min, with general clinical status and body weight also recorded.

**Figure 4:**
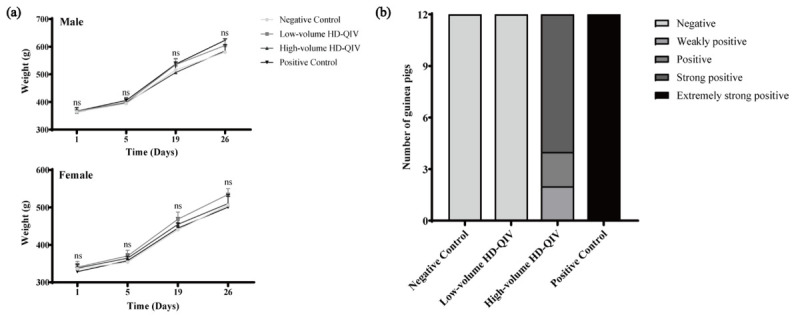
Body weight trend and distribution of allergic reaction intensity in male and female guinea pigs in the HD-QIV systemic allergy test. (**a**) Body weights of male and female guinea pigs measured on Days 1, 5, 19 (*n* = 6), and 28 (*n* = 3) during the sensitization and challenge periods; (**b**) Distribution of anaphylaxis intensity in guinea pigs during the sensitization and challenge periods (*n* = 12). Data are presented as the mean ± SD. ns, *p* ≥ 0.05.

During sensitization and challenge, no animal deaths or general observation abnormalities were noted in any group except the positive control group. Body weights of all animals increased over time, with weight gain trends in male and female guinea pigs from the low-volume, high-volume, and positive control groups being comparable to those in the negative control group (Figure 4a).

Following two challenges, no allergic reactions were observed in the negative control group (0/12), whereas severe allergic reactions occurred in all animals of the positive control group (12/12). The low-volume group tested negative for anaphylaxis (0/12), while the high-volume group presented weak positive (2/12), positive (2/12), and strong positive (8/12) reactions, respectively (Figure 4b). In view of the allergic responses observed in the high-volume group post-challenge, two healthy non-sensitized guinea pigs (one male and one female) per group were intravenously administered with the same HD-QIV dose as used for challenge in the low- and high-volume groups. No allergic symptoms or other abnormalities were observed during observation, excluding potential anaphylactoid reactions directly induced by the vaccine formulation.

### 3.4. Antigen Dose–Response Relationship Study

**Figure 5:**
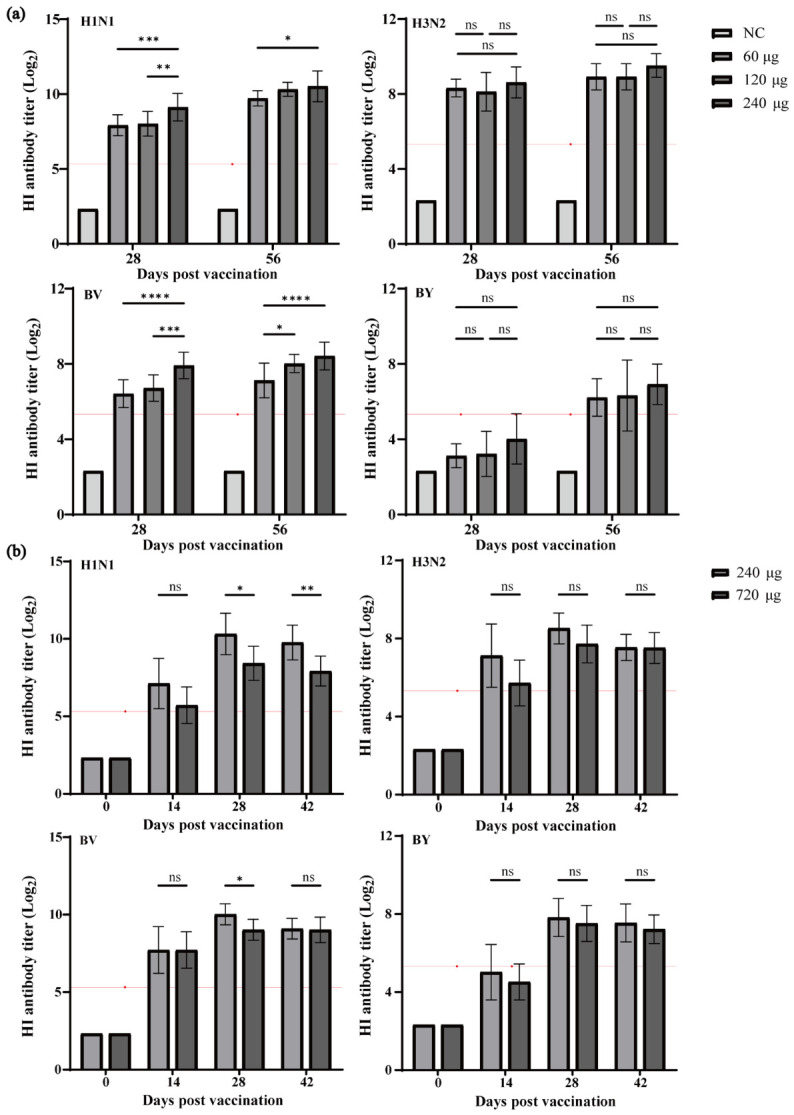
Immunogenicity of Different HD-QIV Doses in BALB/c Mice and SD Rats (*n* = 10). (**a**) HI antibody titers in BALB/c mice on Days 28 and 56 after the first dose; (**b**) HI antibody titers in SD rats on Days 0, 14, 28, and 42 after the first dose. Data are presented as the mean ± SD. The red line with points indicates the Log_2_-transformed value corresponding to a HI antibody titer of 40. ns, *p* ≥ 0.05; *, *p* < 0.05; **, *p* < 0.01; ***, *p* < 0.001; ****, *p* < 0.0001.

Pre-immunization HI titers of BALB/c mice in all groups were 5. On Days 28 and 56 post-immunization, the seroconversion rates for H1N1, H3N2, and BV subtypes reached 100%. For the BY subtype, dose-dependent seroconversion rates were observed on Day 28: 10% (60 μg), 40% (120 μg), and 60% (240 μg), with seroconversion rates increasing to 100% for the 60 μg and 240 μg groups by Day 56 (Table S5). Analysis of HI antibody titers revealed immune responses across different influenza subtypes. For the H1N1 subtype, HI antibody titers in the 240 μg group were significantly higher than those in the 60 μg and 120 μg groups at Day 28 post-vaccination (*** *p* < 0.001, ** *p* < 0.01). At Day 56, the 240 μg group maintained significantly elevated antibody titers compared with the 60 μg group (* *p* < 0.05), and all vaccine dose groups retained robust antibody levels. For the H3N2 subtype, no statistically significant differences in HI antibody titers were observed among the 60 μg, 120 μg, and 240 μg groups at either Day 28 or Day 56 (all ns). For the BV subtype, the 240 μg group exhibited significantly higher HI antibody titers than the 60 μg and 120 μg groups at Day 28 (**** *p* < 0.0001, *** *p* < 0.001), and this group also showed significantly higher titers than the 60 μg group at Day 56 (**** *p* < 0.0001). For the BY subtype, no significant differences in HI antibody titers were detected among the three dose groups at either Day 28 or Day 56 (all ns) (Figure 5a).

All SD rat groups had pre-immunization HI titers of 5. On Day 14 post-immunization, the 240 μg dose group achieved 100% seroconversion for H1N1, 90% for H3N2, 100% for BV, and 60% for BY; the 720 μg dose group showed 80% seroconversion for both H1N1 and H3N2, 100% for BV, and 30% for BY. By Days 28 and 42 post-immunization, all subtypes reached 100% seroconversion in all groups (Table S6). For HI antibody titers, the 240 μg group had significantly higher titers than the 720 μg group for H1N1 on days 28 and 42 (* *p* < 0.05, ** *p* < 0.01), and for BV on day 28 (* *p* < 0.05). No significant differences were observed between dose groups for H3N2 or BY at any time point (all ns) (Figure 5b).

In conclusion, across both animal models, the BY subtype exhibited an overall lower immune magnitude and greater individual variability in seroconversion rates and HI antibody levels compared with the H1N1, H3N2, and BV subtypes. In mice, HI antibody titers increased in a dose-dependent manner. In SD rats, however, further elevation of the antigen dose beyond the HD-QIV dosage did not enhance HI antibody titers; instead, it resulted in concurrent reductions in both antibody titers and seroconversion rates.

### 3.5. Comparative Study with Licensed Vaccine

**Figure 6:**
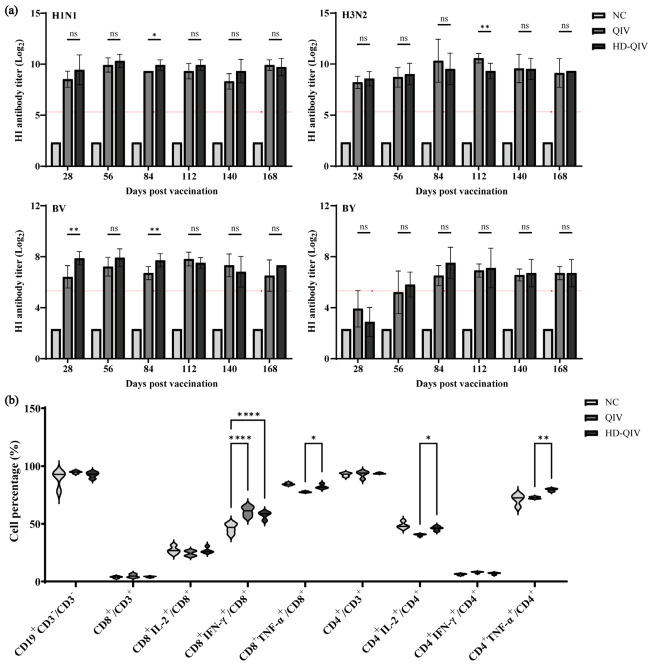
Humoral immune responses at Days 28, 56, 84, 112, 140, and 168 and cellular immune responses at Day 56 after QIV and HD-QIV vaccination in 6–8 week-old BALB/c mice. (**a**) HI antibody titers (*n* = 10); (**b**) Proportions of T cell subsets. Data are presented as the mean ± SD. ns, *p* ≥ 0.05; *, *p* < 0.05; **, *p* < 0.01; ****, *p* < 0.0001. The red line with points indicates the Log_2_-transformed value corresponding to a HI antibody titer of 40.

This study evaluated the immunogenicity of HD-QIV versus QIV in young and aged BALB/c mice. For humoral immunity, all groups exhibited a baseline serum HI antibody titer of 5 prior to immunization. In 6–8 week-old mice, QIV and HD-QIV exhibited comparable seroconversion rates for the H1N1, H3N2, and BV subtypes, with both groups maintaining 100% seroconversion from Day 28 to 168 post-vaccination. For the BY subtype, the HD-QIV group achieved 100% seroconversion by Day 56, compared to 80% in the QIV group, and both groups sustained 100% seroconversion from Day 84. HD-QIV also demonstrated significantly higher HI antibody titers against H1N1 on Day 84, and against BV on Days 28 and 84 (Figure 6a and Table S7). In 16-month-old mice, HD-QIV achieved 100% seroconversion for H1N1 by Day 56, compared with Day 84 in the QIV group, and exhibited significantly higher HI antibody titers on Days 56 and 84. While both groups maintained 100% seroconversion for H3N2 throughout the study, HD-QIV had significantly higher HI antibody titers on Days 56 and 84. For the BV subtype, HD-QIV achieved 100% seroconversion earlier and had a delayed onset of titer decline, along with significantly higher HI antibody titers on Day 84. For the BY subtype, HD-QIV had an extended duration of 100% seroconversion (Days 28–84 vs. Days 28–56 in QIV) and significantly higher HI antibody titers on Day 84 (Figure S3a and Table S8).

In terms of cellular immune responses, HD-QIV induced significantly higher proportions of CD3^+^CD8^+^TNF-α^+^, CD3^+^CD4^+^IL-2^+^, and CD3^+^CD4^+^TNF-α^+^ T cells in 6–8 week-old mice (Figure 6b), whereas no significant differences in T cell subsets were observed between QIV and HD-QIV groups in 16-month-old mice (Figure S3b).

Notably, even after administration of HD-QIV, 16-month-old mice exhibited significantly lower HI antibody titers against H1N1, H3N2, and BV compared with young mice, indicating that HD-QIV did not fully reverse age-related decline in humoral immunity. Furthermore, increasing antigen dosage exerted limited effects on cellular immunity in both age groups, consistent with the dominant role of humoral immunity in the immunological profile of split influenza vaccines.

## 4. Discussion

This study systematically evaluated the safety and immunogenicity of a domestically developed high-dose quadrivalent influenza split vaccine. Compared with imported counterparts such as Fluzone High-Dose Quadrivalent, this candidate is China’s first self-developed and domestically manufactured high-dose quadrivalent seasonal influenza split vaccine with independent intellectual property rights, featuring prominent cost-effectiveness via a local production platform. Currently, no domestically manufactured high-dose influenza vaccine has been approved in China, these results provide critical preclinical evidence for the clinical development and regulatory approval of this candidate vaccine.

Single- and repeated-dose toxicity studies showed that HD-QIV only elicited mild and spontaneously reversible local irritant reactions in SD rats, with no additional adverse reactions observed, and the overall safety profile was consistent with that of quadrivalent influenza vaccine (QIV) [27,28]. Although some groups had statistically significant changes in certain hematological and biochemical parameters, these were minor, non-dose-dependent, and unaccompanied by relevant pathological changes, indicating no association with HD-QIV exposure. Furthermore, anaphylactoid manifestations were observed in the high-volume HD-QIV group, which was identical to the clinical dose, with strong positive reactions recorded in 8 out of 12 animals. Challenge tests in non-sensitized guinea pigs were all negative, confirming that these reactions elicited by repeated sensitization and challenge, rather than non-specific irritation directly induced by HD-QIV. Notably, the high-volume dose represented a supraphysiological level in guinea pigs. After normalization by body weight, the actual antigen exposure was relatively high, indicating that these allergic findings are clinically irrelevant in humans. In addition, type I hypersensitivity in guinea pigs is predominantly IgG-mediated, which is different from the IgE-driven pathway in humans. Thus, the observed reactions were more likely attributable to excessive immune activation, complement activation, or mast cell stimulation under high antigen load, combined with the inherent hypersensitivity of guinea pigs and the effects of the sensitization–challenge regimen, rather than vaccine sensitization. Species differences mean guinea pig findings cannot fully represent human clinical responses, but subsequent clinical trials can ensure safety via dose escalation, rigorous monitoring, and contingency protocols. These results are consistent with the safety profile of the Fluzone High-Dose vaccine and support the further clinical development of HD-QIV [29].

HD-QIV induced high-level HI antibody responses in both BALB/c mice and SD rats, but the dose–immunogenicity relationship was non-linear. Antibody titers increased with antigen dosage in BALB/c mice, whereas in SD rats, the 720 μg group showed lower HI titers than the 240 μg group, indicating that excessively high antigen doses may suppress immune responses. This phenomenon can be explained by several immunobiological mechanisms. First, excess antigen triggers over-crosslinking of B cell receptors to induce B cell anergy or apoptosis, and disrupts germinal center reactions to impair antibody affinity maturation and the generation of long-lived plasma cells. Second, surplus antigen activates regulatory T cells to secrete inhibitory cytokines such as IL-10 and TGF-β, while impairing dendritic cell function and driving tolerogenic transformation, thereby weakening antigen presentation and Th2/B cell-mediated immune responses. In aged mice, HD-QIV exhibited significantly superior immunogenicity compared with conventional QIV, with earlier seroconversion and longer-lasting antibody persistence, which is consistent with clinical results of approved high-dose influenza vaccines, providing experimental evidence for its application in elderly populations [30,31]. Meanwhile, cellular immunity was minimally affected by antigen dose, indicating that HD-QIV predominantly activates humoral immunity, whereas cellular immunity only plays an auxiliary role in the vaccine-conferred immune protection. The mild alterations in CD4^+^/CD8^+^ T-cell cytokines observed in this study further confirm this pattern.

Notably, compared with other influenza subtypes, the BY lineage induced inherently weaker immune responses with more pronounced individual variation in the present study. This phenomenon may be attributed to the intrinsic antigenic characteristics and distinct antigen-processing pathways of BY lineage strains, which tend to confer lower immunogenicity and trigger heterogeneous immune reactivity [32]. Furthermore, the BY lineage elicits delayed and blunted innate immune responses in respiratory epithelial cells, particularly manifested as impaired interferon production and attenuated TSLP expression [33,34]. Such retarded and dampened innate immune activation constrains the subsequent adaptive antibody response. These findings highlight the necessity of targeted optimization for the B lineage component in influenza vaccine formulations to achieve potent and stable immune protection, which is particularly critical for susceptible populations such as the elderly.

Following the near disappearance of the B/Yamagata lineage influenza virus in humans, the WHO has encouraged a gradual transition from quadrivalent to trivalent influenza vaccines. Removal of the B/Yamagata component would streamline vaccine manufacturing and reduce production costs. Nevertheless, the present study was designed and completed in accordance with earlier WHO quadrivalent vaccine recommendations, adhering strictly to GLP-compliant preclinical protocols, and thus provides essential data support for regulatory registration. Furthermore, the temporary absence of the B/Yamagata lineage does not imply permanent eradication; our findings establish a safety and immunogenicity baseline to enable rapid preparedness in the event of its potential re-emergence. For immunosenescent elderly populations, the safety, dose–response, and immunogenicity results generated herein can be directly extrapolated to high-dose trivalent influenza vaccines, supporting the development of domestic high-dose trivalent vaccines and improving vaccine accessibility. This work also provides key mechanistic insights for optimizing the dosage and composition of inactivated influenza vaccines. In summary, the present findings remain valuable for the future development of high-dose trivalent influenza vaccines.

Several limitations of this study should be acknowledged. First, viral challenge experiments were not conducted to directly verify the protective efficacy of HD-QIV, mainly because this study focused on early preclinical safety and immunogenicity assessment. Viral challenge tests are resource-intensive, time-consuming, and ethically constrained, making them more suitable for later-stage preclinical research. Second, although microneutralization assays were not performed, HI antibody titers still represent the most widely accepted serological correlate of protection for influenza vaccines. In addition, only female mice were used for immunogenicity evaluation in this study. Compared with male mice, female mice present a more stable basal immune phenotype and lower inter-individual experimental variation, which can effectively ensure the reproducibility and statistical reliability of experimental data; nevertheless, this experimental design introduces a potential sex bias. These limitations should be considered when interpreting and extrapolating the immunogenicity findings, although these limitations overall do not affect the reliability of the core conclusions and only somewhat restrict the comprehensive evaluation of the functional protective efficacy of HD-QIV.

Compared with similar imported products, the HD-QIV in this study adopts a domestic manufacturing process and presents distinct cost-effectiveness advantages. In addition, considering that the target population of the vaccine is the elderly, reproductive toxicity studies were not conducted in this research. Although HD-QIV partially alleviated the age-related decline in immune responsiveness, antibody levels in aged mice remained significantly lower than those in young mice, suggesting that simply increasing the antigen dose, while beneficial, cannot fully overcome age-related immune senescence. Therefore, the incorporation of adjuvants suitable for immunocompromised populations, such as MF59 and AS01, represents a feasible strategy to further enhance immune responses in the elderly.

## 5. Conclusions

In summary, the HD-QIV demonstrated a favorable safety profile in preclinical studies. After single and repeated intramuscular administration in SD rats, only mild and reversible local irritation was observed, with no evidence of systemic toxicity. In guinea pig studies, the anaphylactoid reactions induced by high antigen doses represented non-specific excessive immune activation unrelated to the typical IgE-mediated allergic mechanism in humans, thus having limited clinical relevance. The HD-QIV elicited robust humoral immune responses and exhibited significantly superior immunogenicity in aged mice, providing a preclinical basis for its further clinical development and application in the elderly population.

## Figures and Tables

**Table 1:** Routine Hematological Parameters and Coagulation Function Indicators of Male and Female Rats in the HD-QIV Repeated-Dose Toxicity Assay.

Sex	Time	Group	MCHC (g/L)	EO%	APTT (s)	FIB (g/L)	TT (s)
Male	Day 15	NC	349 ± 6	1.8 ± 0.6	10.5 ± 1.6	2.36 ± 0.17	30.8 ± 1.0
		Low Dose HD-QIV	348 ± 5	1.5 ± 0.5	11.9 ± 1.2	2.43 ± 0.16	32.5 ± 1.0 **
		High Dose HD-QIV	350 ± 5	1.9 ± 0.6	11.8 ± 0.7	2.66 ± 0.42 *	30.5 ± 4.8
	Day 43	NC	343 ± 3	1.4 ± 0.3	9.0 ± 2.7	2.18 ± 0.16	30.1 ± 3.2
		Low Dose HD-QIV	339 ± 4	2.1 ± 0.9	9.0 ± 0.9	2.10 ± 0.15	29.0 ± 0.9
		High Dose HD-QIV	340 ± 5	2.0 ± 0.4	9.0 ± 1.5	2.09 ± 0.16	31.1 ± 1.1
Female	Day 15	NC	348 ± 3	1.9 ± 0.9	15.8 ± 1.0	2.02 ± 0.10	39.1 ± 3.5
		Low Dose HD-QIV	345 ± 3	2.1 ± 0.7	15.1 ± 0.9	2.03 ± 0.17	37.6 ± 2.7
		High Dose HD-QIV	351 ± 3 *	3.2 ± 1.6 *	14.7 ± 1.0 *	2.19 ± 0.15 *	37.7 ± 1.6
	Day 43	NC	342 ± 2	2.3 ± 0.5	8.7 ± 1.5	1.67 ± 0.13	29.8 ± 1.3
		Low Dose HD-QIV	343 ± 1	3.0 ± 0.4	8.9 ± 1.1	1.90 ± 0.15 *	29.8 ± 1.8
		High Dose HD-QIV	350 ± 5 *	2.1 ± 0.6	9.0 ± 0.7	1.89 ± 0.10 *	30.6 ± 1.7

Only test items exhibiting significant differences relative to the control group are presented. Data are presented as the mean ± SD. *, *p* < 0.05; **, *p* < 0.01 (Day 15 after the first dose, *n* = 10; Day 43 after the first dose, *n* = 5). MCHC, mean corpuscular hemoglobin concentration; EO, eosinophils; APTT, activated partial thromboplastin time; FIB, fibrinogen; TT, thrombin time.

**Table 2:** Serum Biochemical Indices of Male and Female Rats in the HD-QIV Repeated-Dose Toxicity Assay.

Sex	Time	Group	ALB (g/L)	CK (U/L)	AST (U/L)	TCHO (mmol/L)	TG (mmol/L)	CREA (μmol/L)	UREA (mmol/L)	TP (g/L)	Cl^−^ (mmol/L)	GLO (U/L)	A/G
Male	Day 15	NC	40.5 ± 1.6	543 ± 58	97 ± 8	1.62 ± 0.23	0.5 ± 0.2	22 ± 2	5.84 ± 0.83	53.8 ± 2.4	100.6 ± 1.6	13.2 ± 1.7	3.11 ± 0.39
		Low Dose HD-QIV	40.5 ± 1.0	490 ± 130	90 ± 11	1.74 ± 0.25	0.5 ± 0.1	22 ± 2	5.80 ± 0.70	53.0 ± 1.6	100.9 ± 2.2	12.6 ± 1.2	3.25 ± 0.31
		High Dose HD-QIV	40.6 ± 1.5	409 ± 78	97 ± 9	1.97 ± 0.18	0.6 ± 0.2	22 ± 2	5.25 ± 0.35	54.1 ± 1.5	102.1 ± 1.2	13.4 ± 1.2	3.05 ± 0.33
	Day 43	NC	39.2 ± 1.2	615 ± 146	94 ± 12	1.53 ± 0.34	0.5 ± 0.2	24 ± 2	5.56 ± 0.63	56.1 ± 1.7	103.6 ± 1.3	16.9 ± 0.6	2.32 ± 0.07
		Low Dose HD-QIV	41.0 ± 1.4	831 ± 205	107 ± 14	1.47 ± 0.14	0.5 ± 0.1	25 ± 5	6.91 ± 1.19 *	55.2 ± 1.7	103.0 ± 1.2	14.2 ± 1.0 **	2.89 ± 0.24 **
		High Dose HD-QIV	41.7 ± 1.3 *	450 ± 122	83 ± 9	1.54 ± 0.16	0.8 ± 0.3	24 ± 1	5.60 ± 0.38	57.1 ± 1.7	105.2 ± 2.1	15.4 ± 1.8	2.74 ± 0.38 *
Female	Day 15	NC	43.7 ± 1.8	519 ± 164	100 ± 15	1.70 ± 0.30	0.4 ± 0.1	25 ± 2	5.58 ± 0.58	57.9 ± 1.9	101.8 ± 1.1	14.2 ± 1.2	3.11 ± 0.32
		Low Dose HD-QIV	44.0 ± 2.2	371 ± 119	82 ± 9 **	1.88 ± 0.27	0.5 ± 0.1	28 ± 6	5.72 ± 0.99	57.4 ± 2.3	102.3 ± 0.9	13.4 ± 1.2	3.33 ± 0.40
		High Dose HD-QIV	43.4 ± 2.4	398 ± 148	92 ± 16	2.10 ± 0.16 **	0.6 ± 0.1 **	27 ± 2	6.08 ± 0.44	57.4 ± 3.0	102.1 ± 0.9	14.0 ± 1.4	3.12 ± 0.36
	Day 43	NC	46.5 ± 2.9	353 ± 40	78 ± 9	2.09 ± 0.22	0.9 ± 0.7	28 ± 2	5.61 ± 0.36	61.3 ± 2.8	104.3 ± 0.7	14.8 ± 0.8	3.15 ± 0.29
		Low Dose HD-QIV	46.9 ± 2.2	389 ± 135	81 ± 14	2.37 ± 0.31	0.7 ± 0.3	25 ± 2 *	5.28 ± 0.41	65.2 ± 1.3 *	105.5 ± 0.8	18.3 ± 1.2 **	2.58 ± 0.28 **
		High Dose HD-QIV	45.5 ± 1.1	175 ± 19 **	67 ± 6	1.78 ± 0.44	0.5 ± 0.3	28 ± 2	5.47 ± 0.30	62.5 ± 2.0	106.9 ± 1.3 **	17.1 ± 1.3 *	2.67 ± 0.20 *

Only test items exhibiting significant differences relative to the control group are presented. Data are presented as the mean ± SD. *, *p* < 0.05; **, *p* < 0.01 (Day 15 after the first dose, *n* = 10; Day 43 after the first dose, *n* = 5). ALB, albumin; CK, creatine kinase; AST, aspartate aminotransferase; TCHO, total cholesterol; TG, triglycerides; CREA, creatinine; UREA, urea; TP, total protein; Cl^−^, chloride; GLO, globulin; A/G, albumin/globulin ratio.

## Data Availability

The data presented in this study are available on request from the corresponding author.

## Supplementary Materials

The following supporting information can be downloaded at: https://www.mdpi.com/article/10.3390/vaccines14050446/s1, Figure S1: Gating strategy for flow cytometric detection of antigen-specific CD4+ and CD8+ T cell subsets and their intracellular cytokine (IFN-γ, TNF-α, IL-2) expression in mouse splenocytes; Figure S2: Relative organ weights and T lymphocyte subsets in male and female SD rats during HD-QIV repeated-dose toxicity assay. (a) Relative visceral organ weights (wet weight/body weight ratio) on Day 15 (*n* = 10) and Day 43 (*n* = 5) after the first dose; (b) Proportions of CD3+, CD4+, and CD8+ T lymphocytes on Day 15 (*n* = 10) and Day 43 (*n* = 5) after the first dose. Data are presented as mean ± SD; Figure S3. Humoral immune responses at Days 28, 56, 84, 112, 140, and 168 and cellular immune responses at Day 56 after QIV and HD-QIV vaccination in 16-month-old BALB/c mice. (a) HI antibody titers (*n* = 10); (b) Percentages of T cell subsets. Data are presented as the mean ± SD; Table S1: Summary of Preclinical Study Design for HD-QIV, including Study Type, Groups, and Key Endpoints; Table S2: Routine Hematological Parameters and Coagulation Function Indicators of Male and Female Rats in the HD-QIV Repeated-Dose Toxicity Assay on Day 15 (n = 10) and Day 43 (n = 5) After the First Dose; Table S3: Serum Biochemical Indices of Male and Female Rats in the HD-QIV Repeated-Dose Toxicity Assay on Day 15 (n = 10) and Day 43 (n = 5) After the First Dose; Table S4: Routine Urinalysis of Male and Female Rats in the HD-QIV Repeated-Dose Toxicity Assay on Day 15 (n = 10) and Day 43 (n = 5) After the First Dose; Table S5: The Average HI Antibody Titers and Seroconversion Rates of BALB/c Mice in Antigen Dose–Response Relationship Study on Day 28 and 56 After the First Dose (n = 10); Table S6: The Average HI Antibody Titers and Seroconversion Rates of SD Rats in Antigen Dose–Response Relationship Study on Day 0, 14, 28 and 42 After the First Dose (n = 10); Table S7: The Average HI Antibody Titers and Seroconversion Rates of 6–8 week-old BALB/c Mice in Comparative Study on Day 28, 56, 84, 112, 140 and 168 After the First Dose; Table S8: The Average HI Antibody Titers and Seroconversion Rates of 16-month-old BALB/c Mice in Comparative Study on Day 28, 56, 84, 112, 140 and 168 After the first dose.
