# Supplementary material for: Evaluation of Safety and Immunogenicity of High-Dose Quadrivalent Seasonal Influenza Split Vaccine: A Preclinical Study
Source: Vaccines (Basel). 2026 May 17;14(5):446. doi: 10.3390/vaccines14050446 (PMC13211341; doi:10.3390/vaccines14050446)
Supplement: Supplementary file 1 [file vaccines-14-00446-s001.zip › Table S1.pdf]

Table S1: Summary of Preclinical Study Design for HD-QIV, including Study Type, Groups, and Key Endpoints

Study Type	Groups	Key Endpoints & Assessments
Single-dose toxicity (a)	HD-QIV (10♀+10♂)<br>Negative Control (10♀+10♂)	General observations, body weight, temperature, food intake, anatomical examination (Day 15)
Repeated-dose toxicity (b)	High-volume HD-QIV (15♀+15♂)<br>Low-volume HD-QIV (15♀+15♂)<br>Negative Control (15♀+15♂)	Body weight, temperature, local reactions, hematology/biochemistry, coagulation, organ weights, histopathology, CD4/CD8 T cells, cytokines, anatomical examination (Day 15, 43)
Active systemic anaphylaxis (c)	Negative Control (6♀+6♂)<br>Low-volume HD-QIV (6♀+6♂)<br>High-volume HD-QIV (6♀+6♂)<br>Positive Control (6♀+6♂)	Body weight, local/systemic effects, allergic reaction grading (Day 19, 26)
Dose-response relationship (d)	PBS Control (10♀)<br>60 µg Group (10♀)<br>120 µg Group (10♀)<br>240 µg Group (10♀)	Hemagglutination inhibition antibody titers (Day 1, 29, 57)
Cellular and humoral immunity (d)	Negative Control (6-8W/16M per 10♀)<br>HD-QIV (6-8W/16M per 10♀)<br>Positive Control (6-8W/16M per 10♀)	HI titers (Day 1, 29, 57, 85, 113, 141, 169), splenocyte analysis (Day 57)
