# Supplementary material for: Evaluation of Safety and Immunogenicity of High-Dose Quadrivalent Seasonal Influenza Split Vaccine: A Preclinical Study
Source: Vaccines (Basel). 2026 May 17;14(5):446. doi: 10.3390/vaccines14050446 (PMC13211341; doi:10.3390/vaccines14050446)
Supplement: Supplementary file 1 [file vaccines-14-00446-s001.zip › Table S2.pdf]

Table S2. Routine Hematological Parameters and Coagulation Function Indicators of Male and Female Rats in the HD-QIV Repeated-Dose Toxicity Assay on Day 15 (*n*=10) and Day 43 (*n*=5) After the First Dose.

Sex	Time	Group	WBC（10^9/L）	RBC（10^12/L）	HGB（g/L）	HCT（%）	MCV（fL）	MCH（pg）	PLT（10^9/L）	NEUT（10^9/L）
Male	Day 15	NC	6.14±1.55	7.69±0.46	150±7	43.0±2.1	55.9±1.9	19.5±1.0	1234±120	0.86±0.25
		Low Dose HD-QIV	7.51±1.79	7.80±0.40	152±5	43.6±1.6	55.9±1.4	19.5±0.6	1249±87	0.96±0.30
		High Dose HD-QIV	6.39±1.72	7.92±0.38	154±6	44.1±1.6	55.7±1.4	19.5±0.7	1298±110	0.99±0.18
	Day 43	NC	7.78±1.95	8.57±0.37	150±6	43.6±1.9	50.9±1.0	17.5±0.4	1099±87	1.12±0.26
		Low Dose HD-QIV	6.59±0.72	8.75±0.33	151±6	44.5±1.1	50.9±1.0	17.2±0.4	1124±160	1.21±0.40
		High Dose HD-QIV	6.54±1.07	8.76±0.41	153±8	45.0±1.9	51.4±1.3	17.5±0.4	1097±63	0.95±0.29
Female	Day 15	NC	5.24±1.78	8.06±0.31	151±6	43.5±1.7	53.9±1.2	18.8±0.4	1243±104	0.64±0.25
		Low Dose HD-QIV	6.48±2.16	8.08±0.35	151±7	43.8±1.7	54.2±0.7	18.7±0.3	1263±106	0.78±0.43
		High Dose HD-QIV	4.20±1.75	7.72±0.35	147±7	41.9±1.8	54.4±1.3	19.1±0.5	1185±397	0.62±0.26
	Day 43	NC	4.54±0.96	8.25±0.43	146±6	42.8±1.7	51.9±1.3	17.8±0.4	1120±76	0.55±0.13
		Low Dose HD-QIV	3.36±1.24	8.36±0.25	149±5	43.4±1.5	51.9±1.5	17.8±0.5	1248±152	0.47±0.10
		High Dose HD-QIV	4.62±1.31	8.29±0.21	150±3	43.0±0.7	51.9±1.1	18.2±0.6	1139±95	0.92±0.33

LYMPH（10^9/L）	MONO（10^9/L）	EO（10^9/L）	BASO（10^9/L）	NEUT%	LYMPH%	MONO%	BASO%	RET（10^9/L）	RET%	PT（s）
4.56±1.18	0.60±0.21	0.10±0.02	0.01±0.00	14.1±2.7	74.3±3.5	9.7±1.7	0.1±0.1	318.8±39.6	4.16±0.63	7.6±0.2
5.75±1.41	0.68±0.16	0.11±0.03	0.01±0.01	12.7±1.4	76.5±1.7	9.2±1.6	0.1±0.1	328.0±47.0	4.22±0.70	7.6±0.3
4.67±1.62	0.61±0.16	0.12±0.04	0.01±0.00	16.8±6.0	71.7±6.4	9.5±0.9	0.2±0.1	329.5±25.9	4.18±0.45	7.4±0.3
5.97±1.67	0.58±0.19	0.11±0.03	0.01±0.01	14.9±3.4	76.3±3.5	7.3±1.6	0.1±0.1	262.4±17.8	3.07±0.30	7.6±0.6
4.75±0.57	0.48±0.22	0.14±0.07	0.01±0.00	18.3±5.4	72.3±6.8	7.3±2.6	0.1±0.1	252.7±12.9	2.89±0.16	7.3±0.2
4.96±0.84	0.48±0.10	0.13±0.04	0.01±0.01	14.5±3.8	75.9±4.4	7.4±0.6	0.2±0.1	246.7±11.1	2.82±0.21	7.4±0.3
4.13±1.49	0.36±0.15	0.10±0.04	0.01±0.00	12.4±3.3	78.5±4.3	7.1±2.0	0.2±0.1	253.3±48.1	3.14±0.59	7.0±0.2
5.07±1.75	0.50±0.16	0.13±0.04	0.01±0.01	12.2±6.2	77.8±7.6	7.9±1.5	0.1±0.1	233.5±37.6	2.90±0.48	7.0±0.1
3.15±1.40	0.31±0.14	0.12±0.04	0.01±0.01	15.0±4.1	74.4±4.4	7.2±1.2	0.1±0.2	228.4±18.1	2.97±0.25	7.0±0.2

3.55±0.84	0.33±0.05	0.11±0.03	0.01±0.01	12.4±3.0	77.8±3.6	7.3±0.5	0.1±0.1	249.9±28.9	3.04±0.41	7.0±0.2
2.49±1.10	0.30±0.09	0.10±0.03	0.01±0.01	14.9±4.8	72.8±6.1	9.0±1.6	0.2±0.2	284.6±70.2	3.40±0.83	7.0±0.2
3.27±1.00	0.34±0.17	0.10±0.03	0.00±0.01	20.2±6.3	70.6±5.8	7.0±1.5	0.1±0.1	230.0±37.2	2.78±0.47	7.1±0.2

Data are presented as the mean ± SD. WBC, white blood cells; RBC, red blood cells; HGB, hemoglobin; HCT, hematocrit; MCV, mean corpuscular volume; MCH, mean corpuscular hemoglobin; PLT, platelet count; NEU, neutrophils; LYM, lymphocytes; MONO, monocytes; BASO, basophils; RET, reticulocyte; PT, prothrombin time.
