# Supplementary material for: Evaluation of Safety and Immunogenicity of High-Dose Quadrivalent Seasonal Influenza Split Vaccine: A Preclinical Study
Source: Vaccines (Basel). 2026 May 17;14(5):446. doi: 10.3390/vaccines14050446 (PMC13211341; doi:10.3390/vaccines14050446)
Supplement: Supplementary file 1 [file vaccines-14-00446-s001.zip › Table S3.pdf]

Table S3. Serum Biochemical Indices of Male and Female Rats in the HD-QIV Repeated-Dose Toxicity Assay on Day 15 (*n*=10) and Day 43 (*n*=5) After the First Dose.

Sex	Time	Group	ALT（U/L）	GGT（U/L）	ALP（U/L）	BILT（μmol/L）	GLU（mmol/L）	Na^+（mmol/L）	K^+（mmol/L）
Male	Day 15	NC	35±5	0±0	227±47	1.7±0.3	5.13±0.58	138.5±2.3	4.94±0.27
		Low Dose HD-QIV	34±6	0±0	229±39	1.6±0.4	5.19±0.64	138.3±2.4	4.86±0.31
		High Dose HD-QIV	36±7	0±0	267±49	1.5±0.3	5.09±0.36	139.6±0.8	5.07±0.13
	Day 43	NC	34±5	0±0	136±25	1.2±0.3	6.18±0.68	141.5±1.5	5.05±0.17
		Low Dose HD-QIV	33±6	0±0	145±16	1.3±0.1	5.70±0.47	140.9±0.3	5.25±0.17
		High Dose HD-QIV	36±7	0±0	136±35	1.3±0.2	6.18±0.71	142.1±1.6	5.01±0.26
Female	Day 15	NC	32±21	0±0	114±32	1.3±0.4	5.00±0.72	140.1±0.9	4.63±0.13
		Low Dose HD-QIV	27±4	0±0	121±18	1.2±0.2	5.46±0.59	139.5±1.2	4.73±0.29
		High Dose HD-QIV	28±5	0±0	124±35	1.1±0.2	5.40±0.48	140.1±0.9	4.73±0.25
	Day 43	NC	30±3	0±0	74±14	1.0±0.4	6.84±0.41	140.7±0.8	4.60±0.09
		Low Dose HD-QIV	33±9	0±0	84±26	0.9±0.1	6.49±0.76	142.0±1.1	4.89±0.22
		High Dose HD-QIV	28±4	0±0	73±10	1.0±0.3	6.60±0.51	142.5±1.5	4.63±0.23

Data are expressed as the mean ± SD. ALT, alanine aminotransferase; GGT, gamma-glutamyl transferase; ALP, alkaline phosphatase; BILT, bilirubin total; GLU, glucose; NA^+, sodium; K^-, potassium.
