# Supplementary material for: Evaluation of Safety and Immunogenicity of High-Dose Quadrivalent Seasonal Influenza Split Vaccine: A Preclinical Study
Source: Vaccines (Basel). 2026 May 17;14(5):446. doi: 10.3390/vaccines14050446 (PMC13211341; doi:10.3390/vaccines14050446)
Supplement: Supplementary file 1 [file vaccines-14-00446-s001.zip › Table S4.pdf]

Table S4. Routine Urinalysis of Male and Female Rats in the HD-QIV Repeated-Dose Toxicity Assay on Day 15 (*n*=10) and Day 43 (*n*=5) After the First Dose.

Sex	Time	Group	URO	BIL	KET	BLO	PRO	NIT	WBC	GLU	SG	pH
Male	Day 15	NC	Normal：10/10	Neg：10/10	Neg：8/10<br>+/-：2/10	Neg：10/10	Neg：7/10<br>Trace：3/10	Neg：10/10	Neg：6/10<br>+/-：2/10<br>1+：2/10	Neg：10/10	1.010：4<br>1.015：3<br>1.020：2<br>1.025：1	7.0：2<br>7.5：8
		Low Dose HD-QIV	Normal：10/10	Neg：9/10<br>1+：1/10	Neg：6/10<br>+/-：4/10	Neg：10/10	Neg：5/10<br>Trace：3/10<br>1+：1/10<br>2+：1/10	Neg：10/10	Neg：2/10<br>+/-：2/10<br>1+：6/10	Neg：10/10	1.010：2<br>1.015：2<br>1.020：3<br>1.025：3	6.0：2<br>7.0：2<br>7.5：6
		High Dose HD-QIV	Normal：8/10<br>1+：2/10	Neg：8/10<br>1+：2/10	Neg：7/10<br>+/-：3/10	Neg：9/10<br>+/-：1/10	Neg：9/10<br>Trace：1/10	Neg：10/10	Neg：5/10<br>+/-：4/10<br>2+：1/10	Neg：10/10	1.010：3<br>1.015：5<br>1.020：2	≤5.0：1<br>7.0：1<br>7.5：6<br>8.0：2
	Day 43	NC	Normal：5/5	Neg：5/5	Neg：4/5<br>+/-：1/5	Neg：4/5<br>+/-：1/5	Neg：4/5<br>Trace：1/5	Neg：5/5	Neg：3/5<br>1+：2/5	Neg：5/5	1.015：3<br>1.025：1<br>≥1.030：1	7.0：2<br>7.5：3
		Low Dose HD-QIV	Normal：5/5	Neg：5/5	Neg：1/5<br>+/-：4/5	Neg：5/5	Neg：5/5	Neg：5/5	+/-：2/5<br>1+：3/5	Neg：5/5	1.015：1<br>1.020：2<br>1.025：2	7.5：5
		High Dose HD-QIV	Normal：5/5	Neg：5/5	Neg：5/5	Neg：5/5	Neg：5/5	Neg：5/5	+/-：1/5<br>1+：4/5	Neg：5/5	1.020：2<br>1.025：3	7.0：1<br>7.5：4
Female	Day 15	NC	Normal：10/10	Neg：10/10	Neg：10/10	Neg：10/10	Neg：10/10	Neg：10/10	Neg：10/10	Neg：10/10	≤1.005：1<br>1.010：4	6.5：1<br>7.0：1

										1.015：5	7.5：7<br>8.0：1
	Low Dose HD-QIV	Normal：10/10	Neg：10/10	Neg：10/10	Neg：9/10<br>+/-：1/10	Neg：10/10	Neg：10/10	Neg：9/10<br>+/-：1/10	Neg：10/10	1.010：7<br>1.015：2<br>1.020：1	6.0：1<br>7.0：5<br>7.5：4
	High Dose HD-QIV	Normal：10/10	Neg：10/10	Neg：9/10<br>+/-：1/10	Neg：10/10	Neg：10/10	Neg：10/10	Neg：8/10<br>+/-：2/10	Neg：10/10	1.010：1<br>1.015：5<br>1.020：2<br>1.025：1<br>≥1.030：1	6.5：2<br>7.0：3<br>7.5：4<br>8.0：1
Day 43	NC	Normal：4/5<br>1+：1/5	Neg：5/5	Neg：2/5<br>+/-：3/5	Neg：5/5	Neg：5/5	Neg：5/5	Neg：4/5<br>+/-：1/5	Neg：5/5	1.010：2<br>1.015：1<br>1.020：1<br>≥1.030：1	6.0：1<br>6.5：2<br>7.0：1<br>7.5：1
	Low Dose HD-QIV	Normal：5/5	Neg：5/5	Neg：4/5<br>+/-：1/5	Neg：3/5<br>+/-：1/5<br>1+：1/5	Neg：5/5	Neg：5/5	Neg：2/5<br>1+：3/5	Neg：5/5	1.015：3<br>1.020：1<br>1.025：1	6.0：1<br>6.5：1<br>7.5：3
	High Dose HD-QIV	Normal：5/5	Neg：5/5	Neg：5/5	Neg：5/5	Neg：5/5	Neg：5/5	Neg：4/5<br>1+：1/5	Neg：5/5	1.010：2<br>1.015：3	7.5：5

URO, urobilinogen; BIL, bilirubin; KET, ketone body; BLO, blood; PRO, protein; NIT, nitrate; WBC, leukocyte; GLU, glucose; SG, specific gravity.
