# Supplementary material for: Evaluation of Safety and Immunogenicity of High-Dose Quadrivalent Seasonal Influenza Split Vaccine: A Preclinical Study
Source: Vaccines (Basel). 2026 May 17;14(5):446. doi: 10.3390/vaccines14050446 (PMC13211341; doi:10.3390/vaccines14050446)
Supplement: Supplementary file 1 [file vaccines-14-00446-s001.zip › Table S6.pdf]

Table S6. The Average HI Antibody Titers and Seroconversion Rates of SD Rats in Antigen Dose-Response Relationship Study on Day 0, 14, 28 and 42 After the First Dose (*n*=10).

Day	Group	H1N1		H3N2		BV		BY	
		average HI antibody titer	conversion rate (%)	average HI antibody titer	conversion rate (%)	average HI antibody titer	conversion rate (%)	average HI antibody titer	conversion rate (%)
0	240 μg	5.0	0	5.0	0	5.0	0	5.0	0
	720 μg	5.0	0	5.0	0	5.0	0	5.0	0
14	240 μg	222.0	100	222.0	90	356.0	100	49.0	60
	720 μg	76.0	80	76.0	80	268.0	100	28.0	30
28	240 μg	1792.0	100	416.0	100	1120.0	100	272.0	100
	720 μg	424.0	100	256.0	100	576.0	100	212.0	100
42	240 μg	1137.8	100	204.4	100	600.0	100	231.1	100
	720 μg	296.0	100	208.0	100	592.0	100	168.0	100
