# Supplementary material for: Evaluation of Safety and Immunogenicity of High-Dose Quadrivalent Seasonal Influenza Split Vaccine: A Preclinical Study
Source: Vaccines (Basel). 2026 May 17;14(5):446. doi: 10.3390/vaccines14050446 (PMC13211341; doi:10.3390/vaccines14050446)
Supplement: Supplementary file 1 [file vaccines-14-00446-s001.zip › Table S7.pdf]

Table S7. The Average HI Antibody Titers and Seroconversion Rates of 6–8-week-old BALB/c Mice in Comparative Study on Day 28, 56, 84, 112, 140 and 168 After the First Dose.

Day	Group	H1N1		H3N2		BV		BY	
		average HI antibody titer	conversion rate (%)	average HI antibody titer	conversion rate (%)	average HI antibody titer	conversion rate (%)	average HI antibody titer	conversion rate (%)
28	NC	5.0	0	5.0	0	5.0	0	5.0	0
	QIV	367.6	100	296.3	100	85.7	100	15.2	50
	HD-QIV	697.9	100	380.5	100	237.8	100	8.4	14.3
56	NC	5.0	0	5.0	0	5.0	0	5.0	0
	QIV	970.1	100	519.8	100	149.3	100	37.3	80
	HD-QIV	1280.0	100	422.2	100	242.5	100	56.6	100
84	NC	5.0	0	5.0	0	5.0	0	5.0	0
	QIV	640.0	100	1280.0	100	105.6	100	91.9	100
	HD-QIV	970.1	100	735.2	100	211.1	100	183.8	100
112	NC	5.0	0	5.0	0	5.0	0	5.0	0
	QIV	640.0	100	1522.2	100	226.3	100	121.3	100
	HD-QIV	970.1	100	485.0	100	183.8	100	139.3	100
140	NC	5.0	0	5.0	0	5.0	0	5.0	0
	QIV	320.0	100	761.1	100	113.1	100	95.1	100
	HD-QIV	640.0	100	735.2	100	160.0	100	105.6	100
168	NC	5.0	0	5.0	0	5.0	0	5.0	0
	QIV	970.1	100	557.2	100	91.9	100	105.6	100
	HD-QIV	844.5	100	640.0	100	160.0	100	105.6	100

Partial data were excluded due to insufficient blood volume.
