# Supplementary material for: Evaluation of Safety and Immunogenicity of High-Dose Quadrivalent Seasonal Influenza Split Vaccine: A Preclinical Study
Source: Vaccines (Basel). 2026 May 17;14(5):446. doi: 10.3390/vaccines14050446 (PMC13211341; doi:10.3390/vaccines14050446)
Supplement: Supplementary file 1 [file vaccines-14-00446-s001.zip › Table S8.pdf]

Table S8. The Average HI Antibody Titers and Seroconversion Rates of 16-month-old BALB/c Mice in Comparative Study on Day 28, 56, 84, 112, 140 and 168 After the First Dose.

Day	Group	H1N1		H3N2		BV		BY	
		Average HI antibody titer	conversion rate (%)	Average HI antibody titer	conversion rate (%)	Average HI antibody titer	conversion rate (%)	Average HI antibody titer	conversion rate (%)
	NC	5.0	0	5.0	0	5.0	0	5.0	0
28	QIV	60.6	90	130.0	100	26.4	80	60.6	100
	HD-QIV	80.0	90	260.0	100	37.3	80	87.2	100
	NC	5.0	0	5.0	0	5.0	0	5.0	0
56	QIV	121.3	90	91.9	100	49.2	90	183.8	100
	HD-QIV	394.0	100	211.1	100	130.0	100	260.0	100
	NC	5.0	0	5.0	0	5.0	0	5.0	0
84	QIV	130.0	100	74.6	100	40.0	100	30.3	80
	HD-QIV	367.6	100	298.6	100	85.7	100	139.3	100
	NC	5.0	0	5.0	0	5.0	0	5.0	0
112	QIV	105.6	100	121.3	100	34.8	80	23.0	80
	HD-QIV	269.1	100	320.0	100	52.8	100	23.8	50
	NC	5.0	0	5.0	0	5.0	0	5.0	0
140	QIV	113.1	100	183.8	100	23.0	80	17.4	60
	HD-QIV	127.0	100	113.1	100	12.6	40	15.9	40
	NC	5.0	0	5.0	0	5.0	0	5.0	0
168	QIV	91.9	100	121.3	100	13.2	50	13.2	40
	HD-QIV	67.3	75	113.1	100	18.7	80	13.2	40

Partial data were excluded due to insufficient blood volume.
