# Supplementary figures and images for: Evaluation of Safety and Immunogenicity of High-Dose Quadrivalent Seasonal Influenza Split Vaccine: A Preclinical Study
Source: Vaccines (Basel). 2026 May 17;14(5):446. doi: 10.3390/vaccines14050446 (PMC13211341; doi:10.3390/vaccines14050446)

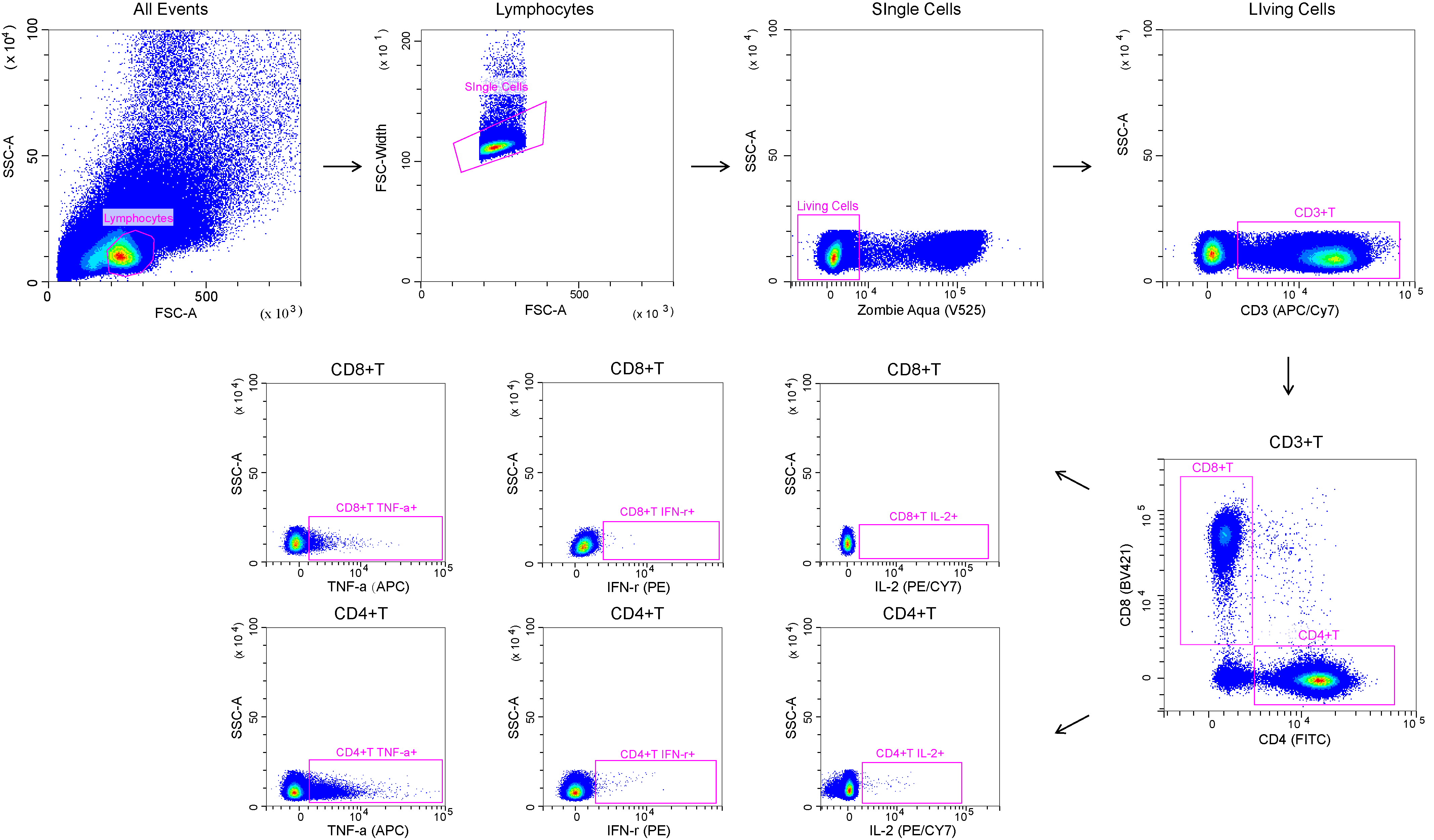

Supplement: Supplementary file 1 [file vaccines-14-00446-s001.zip › Figure S1.tif]
